# Functional Capacity of Tai Chi-Practicing Elderly People

**DOI:** 10.3390/ijerph19042178

**Published:** 2022-02-15

**Authors:** Alba Niño, José Gerardo Villa-Vicente, Pilar S. Collado

**Affiliations:** Institute of Biomedicine (IBIOMED), University of Leon, 24071 Leon, Spain; jg.villa@unileon.es (J.G.V.-V.); mpsanc@unileon.es (P.S.C.)

**Keywords:** aging, physical activity, Tai Chi, Chinese, physical fitness

## Abstract

Research shows that ageing is modifiable or modulable, attending to external modifications and lifestyle factors: physical activity has a unique contribution to functional health and energy balance. Extensive research shows Tai Chi (TC) produced a major physical condition. To determine the impact of lifestyle on functional capacity, comparing the impact of continued long-life practice. 113 individuals (±71.53 years old): (a) PTC (*n* = 27); senior competitors, life-long training; (b) TC (*n* = 27); ±4 years; (c) Keep-Fit (KF *n* = 36); ±4 years; and the control group (d) sedentary individuals (SI *n* = 23). Five tests from the Senior Fitness Test (SFT) were used to assess the physical condition. The TC group showed significantly better results than the KF group: 30-s chair stand (23.22 ± 3.08 * rep vs. 17.17 ± 2.96 rep), chair sit-and-reach (2.19 ± 4.85 * cm vs. −1.93 ± 5.46 cm) and back scratch (1.02 ± 4.46 * cm vs. −2.43 ± 5.78 cm). The TCP group showed better results than the TC group: 30-s chair stand (27.70 ± 4.98 * rep vs. 23.22 ± 3.08 rep), 30-s arm curl (30.22 ± 4.36 * rep vs. 23.48 ± 3.42 rep), chair sit-and-reach (13.07 ± 4.00 * cm vs. 2.19 ± 4.85 cm) and back scratch (5.48 ± 3.51 * cm vs. 1.02 ± 4.46 cm). Among the different activities analysed, TC showed better results in SFT tests; in particular considering the long-life training of this martial art.

## 1. Introduction

Ageing is a life-long process that may imply a progressive loss of physical fitness. Older persons are typically physically inactive and spend more time in sedentary behaviours; it is necessary to emphasise the importance of physical fitness, health and independence for as many years as possible, adapted explicitly to this group [1].

A sedentary lifestyle has detrimental effects and physical consequences for older adults’ health. Inactivity accentuates the progressive loss of autonomy and quality of life of older people at an alarming rate [2,3]. In response to this evidence, many physical activity programs are encouraged by various institutions [4] and extensive research is being conducted by and aimed at public health professionals [5,6,7]. Maintaining fitness capacity (e.g., strength, endurance, agility, and balance) is a key factor in preserving mobility and independence for quotidian activity in later years.

Moreover, research demonstrated that regular physical activity is the only intervention that consistently improves functional health, maintains the energy balance and reduces the risk of lifestyle-related diseases, such as cardiometabolic diseases, obesity, and cerebrovascular diseases [8,9]. Physical activity is also effective in mitigating characteristic diseases such as sarcopenia, restoring physical strength, as well as preventing and delaying the development of disabilities [9]. On the contrary, physical inactivity is clearly related to muscle mass and strength loss: increased physical activity levels should have protective effects as a preventive measure and other lifestyle factors, such as a diet [8,9].

Among the different physical activities recommended for the elderly, Tai Chi is worth studying: Tai Chi is a traditional martial art practiced for defense and health benefits in Chinese society. In Tai Chi, which is a balance-based exercise, slow and rhythmic movements together in a continuous sequence are performed, and the center of gravity (COG) moves with the movements of each foot. Evidence has shown many health benefits of Tai Chi [10], such as relieving psychobiological stress reactivity, promoting psychological wellbeing [11], lowering blood pressure [12], improving flexibility and muscle strength [10], and delaying the results of several chronic diseases [10,13]. The practice of Tai Chi involves lower limb control, lower limb strengthening, and dynamic posture control. People who practice Tai Chi maintain different postures and keep the COG within a changing support base to challenge their balance control system [14].

Another activity offered for this age group is Keep-Fit exercise, referring to a physical and sports activity characterised by low-impact exercises, thus without sudden and fast movements. Muscle workouts are complemented with flexibility, coordination or posture correction [15]. Keep-fit exercise considers the body as a whole: all body parts are equally important, and merge strength, endurance, and joint health. These programmes are focused on health improvements through individualised, safe and motivating exercise aimed at enhancing adherence [16,17,18,19]; the rhythm and expressive components showed differential compliance among women and men [20]: specifically, it seems that women participate in these type of activities to a greater proportion compared to males [20,21,22].

The effects of specific physical exercise programs on older adults have been analysed, but few studies have analysed differences in fitness capacities between different types of fitness programs [23,24]. Studying differences between programs—including its subjectively perceived attractiveness and adherence capacity—may help us to identify or design the most appropriate modalities for older adults and the ones that offer them the most significant benefits. This study aims to (a) compare and assess the functional capacity of older adults who perform supervised activities and a sedentary group (sedentary individuals versus individuals who practise Tai Chi or Keep-Fit exercise), and (b) compare the influence of a recurrent Tai Chi practice in older Chinese adults with an amateur practice of this martial art in the Spanish population.

## 2. Materials and Methods

### 2.1. Participants and Procedures

A cross-sectional descriptive biometric observational study was conducted. The physical condition evaluation was conducted through five Senior Fitness Tests (SFT) in a group of Spanish and Chinese individuals aged 65 or older. The study was designed according to the guidelines of the Declaration of Helsinki, and approved by the Institutional Review Board (or Ethics Committee) of León University (ETICA-ULE-004-2021).

In total, 113 participants (71.53 ± 6.92 years old) were divided into 3 physical activity groups and 1 sedentary control group. Of the Spanish individuals who participate in activities for older adults organised by different city councils, 36 were practising Keep-Fit exercise (KF) (*n* = 30 women and *n* = 6 men), and 27 were practising Tai Chi (TC) (*n* = 21 women and *n* = 6 men), with an average weekly practice of ±3 h over ±4 years, all of them residents in Spain. In total, 27 Chinese residents participants were considered Professional Tai Chi (PTC) athletes due to recurrently practising this sport as a lifestyle with a weekly training of ±12 h and Asian athletes competing in the Senior category (*n* = 12 women and *n* = 15 men). Twenty-three participants corresponded to the sedentary control group (SI) (*n* = 14 women and *n* = 9 men), who were not currently practising or had not practised any physical activity in recent years. (Table 1).

All subjects were informed at a meeting in their town about the evaluation of the study, its procedure, and the records and tests to perform. SFT tests were applied to all participants after the initial warm-up, thus before starting their physical activity, to avoid the effect of fatigue and differences in each group due to their different exercises. Before the competition began, the Asian groups were analysed in a regional championship in Shanghai (China). Vice Dean Ph.D. Zhu Dong of the Shanghai Sports University provided the corresponding license to intervene in this event. The other group was analysed in a senior category exhibition in Shanghai (China). Master Qingquan Fu (6th Generation Master of the Yang style taijiquan and the President of the World Yong Nian Tai Chi Federation) provided the corresponding license to intervene in this event. All participants signed informed consent.

Table 2 shows the characteristics of the exercise programmes performed by the three intervention groups. Keep Fit included a 60-min session with exercises consisting of strength, coordination and agility. The Tai Chi group also performed the routine for 1 h: the first part consisted of structural and postural control, essential positioning and equilibrium; the second part included standardised and characteristic forms; lastly, a flexibility and mobility part was included. The Chinese group, practising long-life professional Tai Chi, divided the activity during the whole day: 60 min in the morning with movements and meditation; 60 min before lunchtime including essential exercise; and a non-predefined time lapse at the end of the day focused on specific forms and flexibility.

### 2.2. Exclusion and Inclusion Criteria

Inclusion criteria were: being 65 years old or older, participating in supervised classes (Keep-Fit exercise or Tai Chi) over 4 years prior to the study or not practising any kind of physical activity regarding the group considered sedentary. The exclusion criterion applied to those who suffered any severe illness or ailment which hinders mobility.

### 2.3. Senior Fitness Test (SFT)

The Senior Fitness Test (SFT) is a battery of seven tests whose purpose is the functional assessment of the physical capacity of the elderly [25]. This evidence-based and validated test has been rectified to evaluate these parameters: upper and lower body strength, upper and lower body flexibility, dynamic balance, agility and aerobic endurance.

The tests carried out consisted of the 30-s chair stand and 30-s arm curl, tests for upper and lower extremity muscle strength, the back scratch and chair sit-and-reach tests for upper and lower body flexibility and the up-and-go test for agility and balance.

### 2.4. Statistical Analysis

The corresponding tests were applied using the statistical software IMB SPSS Statistics 25. The Student’s t-test was applied for independent samples to analyse gender and nationality differences. In order to analyse the mean differences of the SFT tests, the ANOVA statistical analysis of a factor was conducted if normal standards were met in all groups. The Kruskal–Wallis test was used if normal standards were not met within the group.

Regarding post-hoc tests, in the case of the ANOVA’s, the homoscedasticity assumption was not met in all cases, so Welch’s statistical test was calculated. Therefore, the Games Howel statistical test was used as post-hoc testing. The Mann–Whitney U test was used for non-parametric post hoc tests. The values were expressed as mean (SD), and *p* < 0.05 was considered statistically relevant.

## 3. Results

Table 3 shows the anthropometric characteristics of the Spanish sample: a significantly higher BMI—in particular, for women—was observed.

When comparing the Spanish sample with the Chinese sample in terms of anthropometric measures, Chinese participants’ BMI was within normal weight ranges and is significantly lower than the Spanish participants. (Table 4).

Considering the type of physical activity performed, Spanish participants practising Tai Chi have a significantly lower BMI when compared to sedentary individuals and the group that practises Keep-Fit exercise. (Table 5).

Regarding the SFT tests, Table 6 shows that the participants in the study who practice Tai Chi have significantly better values in all tests when analysing the strength, flexibility of arms and legs, and agility compared to sedentary elderly. Consequently, people belonging to the Tai Chi group are in the 100th percentile in the strength and flexibility tests, while the sedentary individuals do not exceed the 50th percentile in any test.

Thus, for the leg strength test, the two active groups, KF and TC, obtained significantly better results than sedentary individuals. The results of those who practice Tai Chi exceed the ones of the KF group, achieving a mean of 23.22 ± 3.08 rep (100th percentile) compared to the 17.17 ± 2.96 rep of the KF group (85th percentile) and the 10.13 ± 1.96 rep of the SI group (25th percentile). Similar results were obtained in the arm strength and agility tests, with values of the TC group in the 100th percentile in both tests and the KF group in the 95th percentile, much higher than the 13.26 ± 2.92 rep (40th percentile) and the 7.28 ± 1.25 s (25th percentile) achieved by the sedentary group in the two tests, respectively.

Significant differences in the TC group were observed in the flexibility tests of both legs and arms compared to the other groups (KF and SI). In the leg flexibility test, the individuals who practise Tai Chi achieve the value of 2.19 ± 4.85 cm (60th percentile) for this test compared to the −1.93 ± 5.46 cm (20th percentile) of the KF group and −4.43 ± 5.88 cm (5th percentile) of the sedentary group. Nevertheless, in the arm flexibility test, the TC group outperformed the participants of the KF and SI groups with 1.02 ± 4.46 cm (80th percentile) again compared to the −2.43 ± 5.78 cm (45th percentile) and −2.94 ± 3.98 cm (40th percentile) achieved by these groups, respectively.

Table 7 shows the comparison of the different SFT variables studied between the two groups practising Tai Chi (amateurs vs. professionals/Spanish vs. Chinese individuals). Both groups have percentiles above 60 in all tests whilst the Chinese people group showed the most optimal values, having the 100th percentile in all tests. The main difference is observed in flexibility tests between Spanish and Chinese. Chinese individuals obtained a leg flexibility mean of 13.07 ± 3.99 cm (100th percentile) compared to 2.19 ± 4.85 cm (60th percentile) achieved by Spanish individuals and a mean of 5.48 ± 3.50 cm (100th percentile) in the arm flexibility test compared to 1.02 ± 4.46 cm (80th percentile) achieved by Spanish individuals. Strictly considering the statistics, Asian participants also outperformed in the strength tests: (a) Asian participants achieved a mean of 30.22 ± 4.36 rep (100th percentile) in the arm strength test, compared to the 23.48 ± 3.42 rep (100th percentile) achieved by the Spanish older adult; and (b) Asian participants achieved 27.70 ± 4.98 rep (100th percentile) in the leg strength test compared to 23.22 ± 3.08 rep (100th percentile) obtained by Spanish participants.

Men and women were compared considering physical activities and absolute values and percentiles. Both men and women who belong to the PTC group have better results in all SFT tests than sedentary individuals and those who practice Keep-Fit exercise and those with an amateur Tai Chi practice (Table 8). Furthermore, although there are significant differences between men and women in this group, all of them fall in the 100th percentile.

No significant differences were found between men and women in the Spanish sedentary group. It should be noted that except in the arm flexibility test, the group is below the 50th percentile in all tests. Likewise, there are no significant differences between men and women in the KF and TC groups, whilst individuals practising Tai Chi had higher percentiles than those in the KF group.

Finally, Table 8 also shows how physical activity practice improved the functional capacities in both men and women regarding sedentary individuals being the continuous practice is the most critical factor concerning those capacities. Thus, to practice Tai Chi for 3 h a week—especially in women—significantly improved leg strength, arm and leg flexibility, and agility.

## 4. Discussion

The novelty of the present research relies on its capacity for unveiling the potential of the Tai Chi practice and long-life physical exercise in terms of functional health, adherence, enjoyment and motivation specifically aimed at seniors.

Research shows that seniors over 70 years of age practising Tai Chi, individualised balance training and exercise control education report an overall improvement in their quotidian activities and their lives in general. In addition, subjects reported greater confidence in balance and movement and changed their usual physical activity to incorporate the continuous practice of TC [26].

Compared to no intervention or other exercises, the present study found that Tai Chi can significantly improve functional mobility and balance in the Tai Chi groups compared to the other groups that received no intervention or other active therapies. Data suggest that when mental and physical control is perceived to be enhanced, with a generalised sense of improvement in overall wellbeing, older persons’ motivation to continue exercising also increases. Diverse research highlighted the TC effects on physical fitness, wellbeing, and general cognition in older adults who practice Tai Chi [27]. Moreover, research showed a psychoemotional outcome related to the development of positive emotions results as well as the emotional connections established in the practice of this martial art [28].

When comparing the group of Spanish individuals who practised Tai Chi or KF with the sedentary group, those practising Tai Chi achieved significantly better results for all SFT tests conducted. In particular, those practising Tai Chi achieved better results: (a) compared to the sedentary individuals; and (b) compared to the results achieved by the KF individuals in leg strength tests and the two flexibility tests. Additionally, those individuals practising KF outperformed the Sedentary Individuals in the two strength and agility tests. Therefore, accordingly to the current research and the literature, these assessed activities would positively affect the performance of basic tasks by the elderly—e.g., sitting down and getting up from a chair or climbing stairs, optimising typical self-care flection movements and the ability to function independently [29]. When comparing these results with similar studies for both genders, older adults who spend more than 4 h a day sitting down (Sedentary Individuals) have worse results in balance, agility, walking speed, strength and aerobic endurance tests [30,31,32]. The improved muscle strength among the participants in the interventions groups is gripping, considering that muscle strength naturally may decline with age. Maintenance of muscle strength may prevent loss of functional dependence [33]. A systematic review reported that exercise might prevent falls and fall-related fractures and reduce risk factors for falls in individuals with low bone mineral density [33].

The current research demonstrated that individuals practising Tai Chi have a better physical condition than those who practised KF or were sedentary. These results are aligned with the literature: significant improvements were obtained in static and dynamic balance, flexibility, and lower and upper body strength tests, as well as improvements in cardiovascular endurance with a Tai Chi practice intervention [34]. Different non-randomised controlled studies and observational studies also assessed this martial art to treat body balance disorder or fall prevention, suggesting that its practice may improve body balance in older people [35] and an added increase in cognitive function [36]. These findings indicate that a short-term and intensive physical training program improves the lower body physical function: for instance, dynamic balance and leg strength and enhances the upper body physical function, fine motor control, and strength [37,38]. These data are also higher for the individuals practising Keep-Fit exercise, as seen in other studies [39], proving that this activity also improves the physical condition in older people when comparing them with sedentary people. Still, our study demonstrated an enhanced physical condition due to practising Tai Chi compared to KF.

Nevertheless, there are differences in our study group when compared with the results obtained in similar samples. Another study [40] including a sample of Spanish people over 80 was compared with the American SFT reference values: better strength and agility results and lower flexibility and resistance levels were observed in the Spanish individuals than in the reference American population, not showing significant differences between genders in the Spanish population. In turn, this fact can also be observed in the study conducted [41] between Spanish and Serbian older women who practised different physical activities: Serbian women showed better physical fitness, upper and lower body flexibility, agility and aerobic endurance, i.e., better levels of physical condition and quality of life than the Spanish women, differences attributed to the type of physical activity performed by both groups.

Consistent physical training improves all aspects studied regardless of the participant’s age: the present research shows how older people who have practised Tai Chi almost all their lives and who, at the time of our study, were training for more than 10 h a week, have a significantly higher physical capacity in all physical characteristics studied.

Similar research studying eventual differences in physical fitness and health in Asian and European older adults shows significantly higher performance levels in motor skills—i.e., aerobic fitness, strength and flexibility—for the Asian sample, aligned the significantly higher results achieved by Chinese individuals in the strength and flexibility tests conducted in our study [42]. Other authors [43] revealed similar differences by observing a consistently lower incidence of self-reported falls in Chinese older people than in Caucasian older people [44,45]. Therefore, a greater understanding of the health, behavioural and lifestyle factors that influence fall rates in Chinese populations is required to elucidate fall prevention strategies in older people. Older Chinese people frequently perform physical activities with low and medium intensity, and maintaining an active lifestyle is a characteristic of this population thus the better physical fitness of the Chinese group assessed in our study may be related to practising Tai Chi throughout their lives [46].

Along these lines, policies aimed at promoting physical exercise and sports throughout the whole lifecycle are needed in order to reduce the impact of the ageing process in modern occidental societies and lessen the health inequalities caused by the lack of access to sports facilities for the most vulnerable populations [47,48]. Our research suggests that Tai Chi and other non-conventional sports and martial arts can be adapted and delivered to senior and older populations and the whole society, offering an attractive and effective alternative in terms of healthy ageing, functional health and socialization [49,50].

## 5. Limitations and Future Research Lines

The present research showed an analysis of the physical condition and functionality of one of the most offered activities aimed at older persons, the Keep Fit, compared, as a feasible alternative, with Tai Chi. The innovation of the study relies on the possibility of studying the effects of a different lifestyle—i.e., the Chinese population—and the long-life and continued physical activity, introducing an international sample of Chinese subjects practising TC. The potential influence of the ethnicity and ethnic factors should be further studied and will be assessed in future research. Considering that the present research compares groups with very different profiles—i.e., Tai Chi professionals, older people who exercise regularly, or sedentary persons—it is recommended for future research to keep track of each training and quality of life of each sample. Whilst the improvement of health indicators with the practice of physical activity and specifically of physical condition with Tai Chi seems to be demonstrated. It is also considered necessary to address the exercise recommendations for this population considering the structural co-determinants: spaces, infrastructures, affordability and opportunities to socialise, enjoy and feel heard and seem should be integrated within the general research and the public health programmes.

## 6. Conclusions

The present research shows how the practice of a physical activity aimed at older persons significantly improves their physical condition. The practice of Tai Chi stands out as physical activity compared to Keep-Fit or physical inactivity, significantly improving physical condition for this age group. Considering the variety of physical activities recommended for seniors, Tai Chi outperforms Keep-Fit in the results, particularly within the SFT tests outcomes. Specifically, long-life training of Tai Chi—as it is being practised among the Chinese population—shows even more significant improvements in leg and arm strength, upper and lower body flexibility, and agility. Our study reviewed effects on seniors’ well-being and general cognition and the development of positive emotions with the practice of Tai Chi. Therefore, there is a need to generate policies and strategies for promoting sports and physical activity to reduce the ageing, adverse health and functionality consequences, considering and tackling the structural co-determinants involved.

## Figures and Tables

**Table 1:** Sample size and distribution in the studies and analyses carried out.

Type of Activity	Women	%	Men	%	No. of Individuals	% of Individuals
Keep-Fit exercise (KF)	30	38.96	6	16.67	36	31.86
Tai Chi (TC)	21	27.27	6	16.67	27	23.89
Professional Tai Chi (PTC)	12	15.58	15	41.67	27	23.89
Sedentary Individuals (SI)	14	18.18	9	25.00	23	20.35
Total	77		36		113	

**Table 2:** Characteristics of programs developed by the three active groups.

	KF	TC	PTC
Hours training per week	±3	±3	±10
Training days per week	±3	±3	±10
Years of practice	±4	±4	Lifetime
Characteristics of physical exercise programs	-Warm up -Upper and lower body strength -Agility and aerobic endurance	-Basic TC exercises -Body structure -Static and dynamic balance -Postural strength and control -Form training -Upper and lower body flexibility	-Morning chikung -Half day session: basic TC exercises, body structure, static and dynamic balance, postural strength and control -Afternoon session: forms training, upper and lower body flexibility

Where KF = Keep-Fit exercise, TC = Tai Chi, PTC = Professional Tai Chi.

**Table 3:** Anthropometric differences in Spanish individuals according to gender.

Anthropometric Data		
	Women	Men
Age (years)	72.51 ± 7.43	71.52 ± 7.74
Weight (kg)	65.96 ± 11.78	74.40 ± 8.06
Height (cm)	157.39 ± 6.51	169.07 ± 4.21
BMI (kg/m^2^)	26.56 ± 4.38 *	26.00 ± 2.28

Mean values ± SD. * = *p* < 0.05.

**Table 4:** Anthropometric differences between Spanish and Chinese individuals.

Anthropometric Data		
	Spanish	Chinese
Age (years)	73.00 ± 7.51	67.59 ± 3.06
Weight (kg)	68.02 ± 11.53	64.68 ± 8.34
Height (cm)	160.24 ± 7.84	166.85 ± 6.49
BMI (kg/m^2^)	26.42 ± 3.96 *	23.19 ± 2.30

Mean values ± SD. * = *p* < 0.05.

**Table 5:** Anthropometric differences according to the type of physical and/or sports activity.

	Age (Years)	Weight (kg)	Height (cm)	BMI (kg/m^2^)
SI	78.91 ± 9.01	70.69 ± 13.16	164.30 ± 7.77	26.09 ± 4.08
KF	71.25 ± 6.01	69.94 ± 9.15	158.55 ± 8.18	27.87 ± 3.70
TC	70.30 ± 4.94	63.18 ± 11.84	159.03 ± 6.30	24.77 ± 3.60 *

Mean values ± SD. * = *p* < 0.05 regarding Keep-Fit exercise. Where SI = Sedentary Individuals, KF = Keep-Fit exercise and TC = Tai Chi.

**Table 6:** Differences in SFT tests according to the type of physical activity performed.

**Tests**	**Group Activity**	
30-s chair stand (rep)	Sedentary Individuals	10.13 ± 1.96
	Keep-Fit	17.17 ± 2.96 ^#^
	Tai Chi	23.22 ± 3.08 ^#$^
30-s arm curl (rep)	Sedentary Individuals	13.26 ± 2.92
	Keep-Fit	21.19 ± 3.45 ^#^
	Tai Chi	23.48 ± 3.42 ^#^
Chair sit-and-reach (cm)	Sedentary Individuals	−4.43 ± 5.88
	Keep-Fit	−1.93 ± 5.46 ^¥^
	Tai Chi	2.19 ± 4.85 ^#^
Back scratch (cm)	Sedentary Individuals	−2.94 ± 3.98
	Keep-Fit	−2.43 ± 5.78 ^¥^
	Tai Chi	1.02 ± 4.46 ^#^
Up-and-go (s)	Sedentary Individuals	7.28 ± 1.25
	Keep-Fit	4.64 ± 0.83 ^#^
	Tai Chi	4.14 ± 0.46 ^#^

Mean values ± SD. ^#^ = *p* < 0.05 regarding sedentary individuals. ^$^ = *p* < 0.05 regarding Keep-Fit exercise. ^¥^ = *p* < 0.05 regarding Tai Chi. Where rep = number of repetitions.

**Table 7:** Influence of professional vs. amateur Tai Chi practice on SFT tests.

**Tests**	**Mean Values ± SD**	
30-s chair stand (rep)	Spanish	23.22 ± 3.08
	Chinese	27.70 ± 4.98 ^¥^
30-s arm curl (rep)	Spanish	23.48 ± 3.42
	Chinese	30.22 ± 4.36 ^¥^
Chair sit-and-reach (cm)	Spanish	2.19 ± 4.85
	Chinese	13.07 ± 4.00 ^¥^
Back scratch (cm)	Spanish	1.02 ± 4.46
	Chinese	5.48 ± 3.51 ^¥^
Up-and-go (s)	Spanish	4.14 ± 0.46
	Chinese	3.91 ± 0.33

Mean values ± SD. Differences in Spanish vs. Chinese individuals: ^¥^ = *p* < 0.05. Where rep = number of repetitions.

**Table 8:** Mean values in percentiles of the different physical activities and the sedentary group for SFT tests.

SFT Tests	Mean Value (Percentile)				
		SI	KF	TC	PTC
30-s chair stand (rep)	♂	10.56 ± 1.81 (20)	19.83 ± 2.85 * (85)	20.83 ± 1.72 * (100)	28.40 ± 4.48 *^ab^ (100)
	♀	9.86 ± 2.07 (15)	16.63 ± 2.72 * (80)	23.90 ± 3.06 *^a^ (100)	26.83 ± 5.62 *^a^ (100)
30-s arm curl (rep)	♂	13.56 ± 1.33 (25)	21.67 ± 3.93 * (85)	22.67 ± 3.72 * (85)	31.20 ± 3.00 *^ab^ (100)
	♀	13.07 ± 3.64 (40)	21.10 ± 3.40 * (95)	23.71 ± 3.39 * (100)	29.00 ± 5.52 *^ab#^ (100)
Chair sit-and-reach (cm)	♂	-6.88 ± 5.23 (10)	−5.83 ± 6.67 (10)	−4.33 ± 4.88 (25)	12.73 ± 4.02 *^ab^ (100)
	♀	−2.85 ± 5.90 (15)	−1.15 ± 4.94 (25)	4.04 ± 2.87 *^a^ (75)	13.50 ± 4.09 *^ab^ (100)
Back scratch (cm)	♂	−4.84 ± 4.19 (55)	−5.08 ± 4.86 (45)	−4.91 ± 3.77 (55)	6.26 ± 3.75 *^ab^ (100)
	♀	−1.72 ± 3.44 (50)	−1.90 ± 5.87 (50)	2.71 ± 2.95 *^a^ (80)	4.50 ± 3.03 *^a^ (95)
Up-and-go (s)	♂	6.51 ± 1.31 (25)	4.31 ± 1.10 * (80)	4.28 ± 0.46 * (80)	4.02 ± 0.21 * (85)
	♀	7.77 ± 0.96 (15)	4.70 ± 0.77 * (80)	4.10 ± 0.45 *^a^ (90)	3.76 ± 0.39 *^a#^ (95)

Mean values ± SD. * = *p* < 0.05 regarding sedentary individuals. ^a^ = *p* < 0.05 regarding Keep-Fit exercise. ^b^ = *p* < 0.05 regarding Tai Chi. ^#^ = *p* < 0.05 gender differences according type of activity. Where SI = Sedentary Individuals, KF = Keep-Fit exercise, TC = Tai Chi, PTC = Professional Tai Chi and rep = number of repetitions.

## Data Availability

Not Applicable.
